# Assessment of Paranasal Sinus Growth with 3D Volumetric Measurements and the Effect of Anatomic Variations on Sinus Volume in a Pediatric Population

**DOI:** 10.3390/tomography12020015

**Published:** 2026-01-26

**Authors:** Ercan Ayaz, Irem Kavukoglu, Nazli Gulsum Akyel

**Affiliations:** 1 Pediatric Radiology Division, Department of Radiology, Başakşehir Çam & Sakura City Hospital, İstanbul 34480, Türkiye; 2 Department of Radiology, Başakşehir Çam & Sakura City Hospital, İstanbul 34480, Türkiye

**Keywords:** paranasal sinus, Agger Nasi cell, concha bullosa, Haller cell, Onodi cell, maxillary sinus, ethmoid sinus, frontal sinus

## Abstract

Background: We aimed to determine paranasal sinus volumes using 3D volumetric measurements and to evaluate the effect of anatomical variations on these volumes, ensuring balanced age and sex distribution during childhood. Methods: Thirteen age groups (0–16 years), each including 10 males and 10 females, were formed. After excluding sinus pathologies, a total of 260 subjects were randomly selected from CT head examinations. Right and left frontal, maxillary, and sphenoid sinus volumes were calculated using 3D Slicer software (version 5.6.2) following manual segmentation of axial CT slices. Also, the presence of right and left Agger Nasi cells, Haller cells, Onodi cells, and concha bullosa were recorded. Results: No significant difference was found between males and females in sinus volumes (*p* > 0.05). Mean right and left maxillary sinus volumes were 6.23 cm^3^ and 6.27 cm^3^ (*p* = 0.551); frontal sinuses were 0.79 cm^3^ and 0.86 cm^3^ (*p* = 0.170); and sphenoid sinuses were 1.64 cm^3^ and 1.85 cm^3^ (*p* = 0.041). Sphenoid sinus pneumatization appeared in 30% of the 0–6-month group and in over 75% of older groups. Frontal pneumatization began at age 2–3 and exceeded 50% after age 4. Agger Nasi, Haller, Onodi cells, and concha bullosa were detected in 58.8%, 31.2%, 10%, and 22.3% of cases, respectively. Anatomical variations showed no significant effect on sinus volumes (*p* > 0.05). Conclusions: We developed a paranasal sinus volume chart applicable to routine practice, showing that anatomical variations had no significant impact on the development. This is the first study to investigate the impact of anatomical variations on sinus development and volume, along with the age at which variations emerge, with a balanced distribution of age and sex.

## Simple Summary

This study is the first article to investigate the impact of anatomical variations on sinus development and volume by 3D segmentations, along with the age at which variations emerge, with a balanced distribution of age and sex. Our volume calculations showed no significant difference between right and left paranasal sinus volumes, between sexes or regarding presence or absence of sinonasal variations. Therefore, we developed a paranasal sinus volume chart suitable for routine practice. Since anatomical variations had no significant impact on the volumes, we believe this chart can be used in all cases.

## 1. Introduction

The paranasal sinuses (PNS) begin to form in utero around the 25th to 28th week of gestation, and their development continues postnatally, with morphological changes extending into adulthood [1]. Among them, the maxillary sinuses are the first to develop and present at birth, whereas the sphenoid sinus pneumatization usually initiates around two years of age, progresses until approximately five years, and may reach its complete formation between fifteen and thirty years of age [2]. In contrast, the frontal sinuses are not present at birth, representing the last of the paranasal air cells to appear, and become detectable on computed tomography (CT) images only between three and five years of age [3]. The ultimate size and shape of the sinuses demonstrate considerable inter-individual variation and depend particularly on the age, sex, and ethnicity of the individual [1,4].

In parallel to normal pneumatization, a wide spectrum of sinonasal anatomical variations has been described, particularly among the ethmoidal air cells. The most frequent anatomic variation is Agger Nasi cells (ANs) (the most anterior ethmoidal air cells) [5,6,7,8]. While some studies found an association between the ANs and frontal sinusitis or mucous retention cysts due to their proximity to the frontal recess [5,6], other investigations found no statistically significant association [7]. Haller cells are located under the inferomedial orbital wall. Several studies showed that the presence of Haller cells was associated with recurrent maxillary sinusitis and mucosal disease [5]. Onodi cells represent the most posterior ethmoidal air cells, above the sphenoid sinus, and are adjacent to the optic nerve. Although these cells are less frequent than other variants, they are of particular surgical importance because of their close relation to the vascular and neural structures [9]. Concha bullosa is defined as pneumatization of the middle turbinate and may be present in 14–67.5% of the population [10]. Its effect on turbinate morphology has been implicated in obstruction of the sinus drainage pathways and may predispose to chronic sinusitis or volume loss, especially in the maxillary sinuses [10].

While the volumetric assessment of PNS has received significant attention in the previous literature, the published studies highlight substantial differences in sinus volumes, measurement techniques (linear, area, or volumetric methods), age and sex distributions, and the reported frequencies of anatomical variations among populations [1,2,3,4,5,6,7,8,9]. Although the clinical significance of PNS variations has been explored in the context of sinusitis and surgical planning, there remains a paucity of data addressing specifically how these variations affect sinus volume, especially during the critical phase of childhood development. Additionally, many studies suffer from non-standard or uneven age groupings and a lack of balanced sex distribution, or are restricted to clinical populations rather than healthy cohorts, thus limiting the establishment of robust normative reference data [1,7,8,11,12]. To our knowledge, there is no previous study that specifically investigated the age at which these anatomical variations first appear in relation to PNS development.

Our institution is the largest hospital complex in Istanbul, and a daily average of 31.1 and 33.6 CT head examinations were performed on patients younger than 16 years old at the pediatric emergency and pediatric clinics, respectively, in 2024. Therefore, our center was able to draw on a substantial pediatric CT head dataset to evaluate variations in the PNS development in children.

The aim of this study was to evaluate the PNS volumes from CT head examinations using three-dimensional volumetric measurements and to investigate the effect of anatomical variations, including ANs, Haller cells, Onodi cells, and concha bullosa, on these volumes in the developing sinuses of children, providing a balanced distribution according to sex and age.

## 2. Materials and Methods

### 2.1. Study Design and Group

This retrospective study was approved by the Clinical Research Ethics Committee of Basaksehir Cam and Sakura City Hospital (protocol code 2024-160). We included pediatric patients (<17 years) who underwent CT head at our institution between 1 January 2023, and 1 August 2024. Inclusion criteria were age below 17 years and CT examinations performed for non-paranasal, non-orbital, and non-maxillofacial indications (e.g., headache, seizure, trauma) justified by pediatricians. Exclusion criteria included poor image quality due to motion or photon starvation artifacts (commonly caused by parental hand positioning), presence of mucosal thickening, retention cysts, or any sinonasal pathology, such as nasal stenosis, choanal atresia, or cystic fibrosis, and a history of sinonasal or maxillofacial surgery or trauma. All procedures adhered to the Strengthening the Reporting of Observational Studies in Epidemiology (STROBE) guidelines [13].

**Figure 1:**
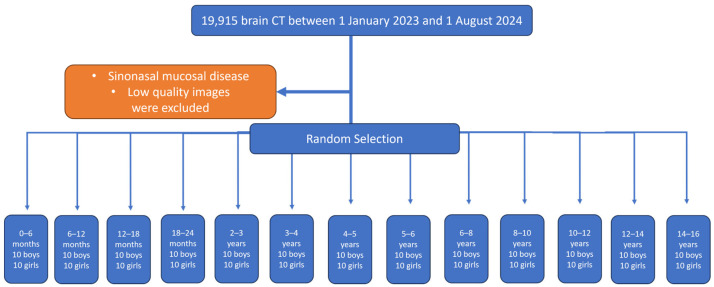
Flowchart of the study and case selection.

The age groups were determined based on the age intervals used in previous studies and the expected volumetric changes between consecutive age groups, while maintaining a balanced female-to-male ratio and an even case distribution (0–2 years in 6-month intervals, 2–6 years in 1-year intervals, and over 6 years in 2-year intervals), resulting in a total of 13 age groups [14]. The upper age limit was set at 16 years, as previous studies have demonstrated that the maxillary sinus reaches its maximal size in both sexes around this age, and our preliminary observations indicated that the frequency of all anatomic variants begins to plateau thereafter [11,15,16,17,18]. The flowchart of study group selection is shown in Figure 1. A total of 19,915 CT head examinations were retrieved and distributed as follows; 1461 in group 1 (0–6 months), 1132 in group 2 (6–12 months), 1170 in group 3 (12–18 months), 1123 in group 4 (18–24 months), 1531 in group 5 (2–3 years), 1427 in group 6 (3–4 years), 1403 in group 7 (4–5 years), 1353 in group 8 (5–6 years), 2207 in group 9 (6–8 years), 2003 in group 10 (8–10 years), 1670 in group 11 (10–12 years), 1557 in group 12 (12–14 years), and 1678 in group 13 (14–16 years). From each group, to achieve 80% statistical power at a 5% significance level (α = 0.05), 10 female and 10 male subjects were randomly selected for each group by using a systematic random sampling method, sampling every fifth case.

### 2.2. CT Acquisition and Volumetric Measurements

The studies were performed using one of four CT scanners within the hospital complex (three 128-slice MDCT systems: Supria 128, Fujifilm Healthcare Co., Tokyo, Japan; and one 128-slice MDCT system: Ingenuity CT, Philips Medical Systems, Best, The Netherlands). Standardized imaging parameters were applied as follows: collimation of 0.625 mm, table speed of 23.8 mm/s, matrix size of 512 × 512, tube voltage of 120 kV, and tube current automatically modulated using automated exposure control (AEC). Images were reconstructed with a 1-mm slice thickness parallel to the temporal bone. The field of view (FOV) ranged from 20 × 20 cm to 28 × 28 cm depending on head size. To allow sharper delineation of bony contours, images were reconstructed using a high-resolution bone kernel algorithm. Based on these axial images, coronal and sagittal multiplanar reformatted images were manually realigned along the true head axis by a radiologist using the institutional PACS system.

**Figure 2:**
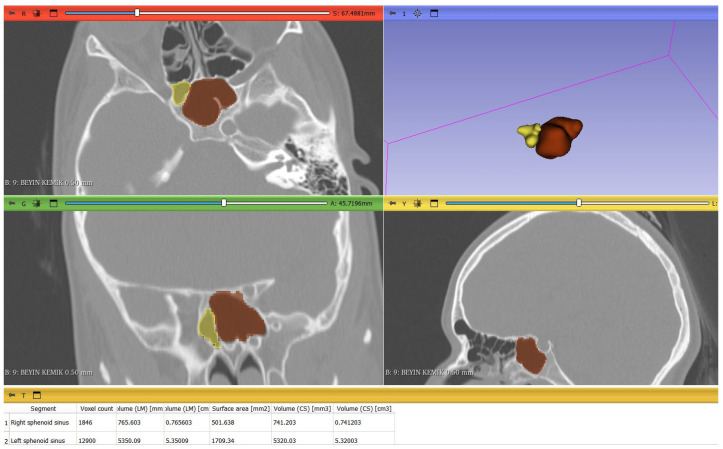
Right (yellow) and left (red) sphenoid sinus segmentation and automatic volume calculation with 3D Slicer^®^ software in a 13-year-old girl.

**Figure 3:**
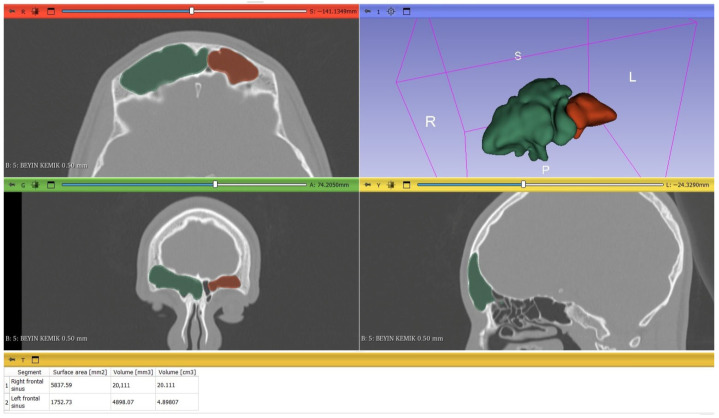
Right (green) and left (red) frontal sinus segmentation and automatic volume calculation with 3D Slicer^®^ software in a 14-year-old boy.

The presence of right and left maxillary, sphenoid (Figure 2), and frontal sinus (Figure 3) and their volume measurements were evaluated using volumetric segmentation by meticulously drawing the margin of PNS, excluding the outflow tracts in each slice of one-millimeter thickness, contiguously from the axial plane images in the 3D Slicer^®^ software. To ensure precise boundary definition, segmentation was conducted on magnified axial images by carefully tracing the inner cortical margins of the hyperdense bony walls. Once the right and left side of each sinus was manually traced craniocaudally, the 3D models of each paranasal air sinus were reconstructed. All segmentation procedures were performed by a single radiologist with four years of experience in maxillofacial CT imaging, who was blinded to the patients’ demographic data, including age and sex. Subsequently, all segmentations were reviewed by a second radiologist with 11 years of experience in maxillofacial CT imaging prior to volumetric analysis. Volumes of each sinus were then automatically calculated by 3D Slicer.

After completion of the volumetric measurements, when a subgroup did not demonstrate a normal distribution (*p* < 0.05), statistical outlier was excluded and replaced with a new case according to the same systematic random sampling approach to preserve subgroup size. This iterative process was continued until a normal distribution was achieved for each subgroup.

**Figure 4:**
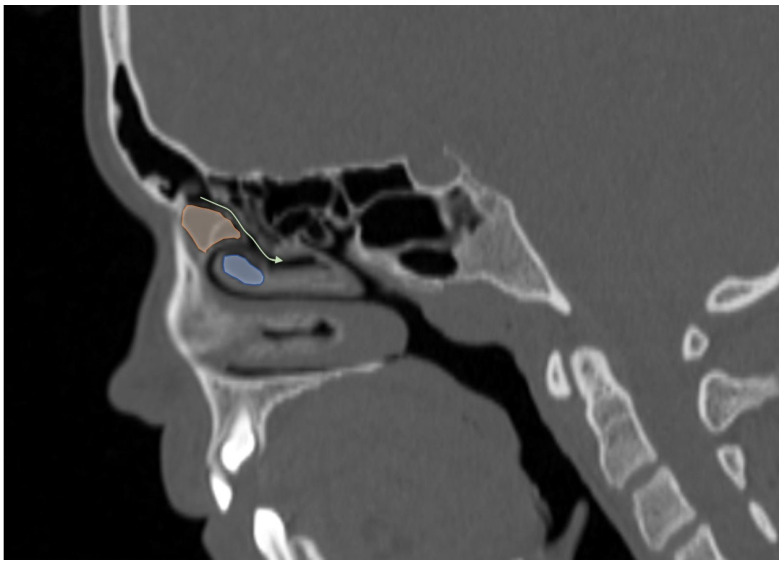
Sagittal reformatted CT image of a 14-year-old girl showing Agger Nasi cell (orange area) in front of the frontal recess draining into the middle meatus (curved arrow) and concha bullosa (blue area).

**Figure 5:**
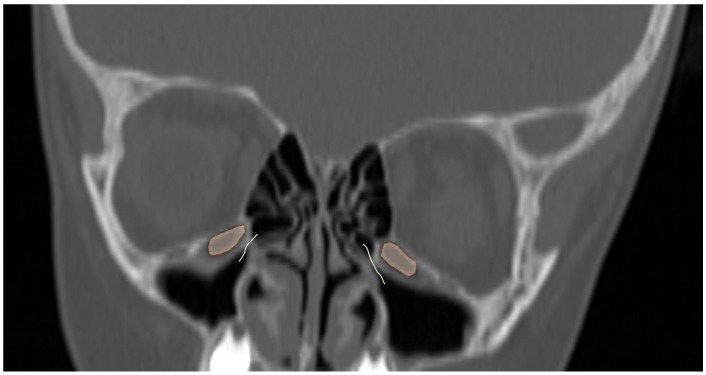
Coronal reformatted CT image of a 6-year-old girl with bilateral Haller cells (orange areas) narrowing the osteomeatal complex (curved lines).

**Figure 6:**
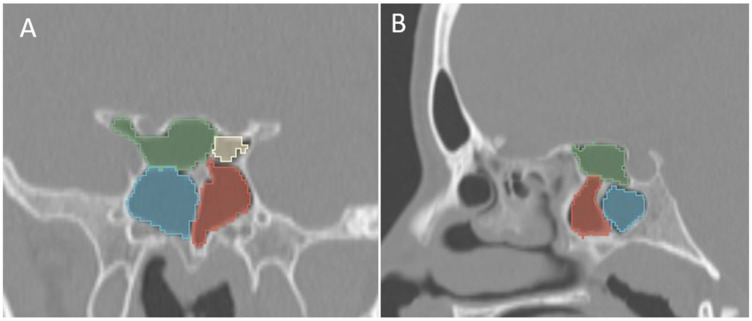
Coronal (**A**) and sagittal (**B**) reformatted CT images of a 13-year-old boy with bilateral Onodi cells (green and yellow) above the sphenoid sinuses (blue and red).

**Figure 7:**
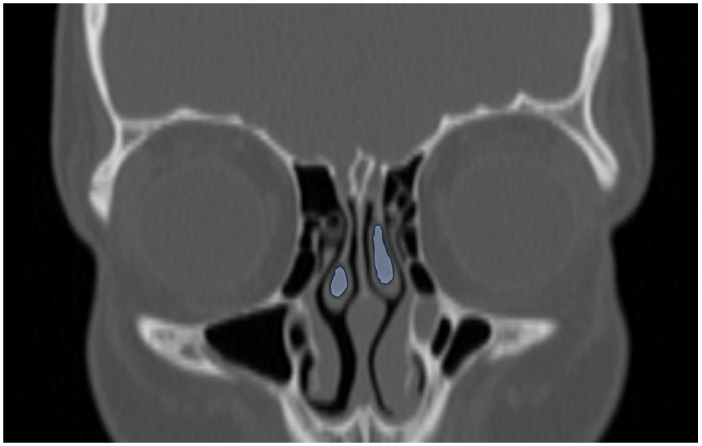
Coronal reformatted CT image of a 12-year-old boy demonstrating bilateral concha bullosa in the middle turbinate (blue areas).

Additionally, the images were assessed for the presence of anatomic variants of the nasal cavity and PNS. Sinonasal variations were defined based on the European Position Paper on the Anatomical Terminology of the Internal Nose and Paranasal Sinuses [19]. According to this paper, the maxillary sinus is located between the orbital floor and alveolar process of the maxilla; the sphenoid sinus is the pneumatization of the sphenoid bone posterior to the rostrum; and the frontal sinus is defined as the pneumatization superior to a noticeable frontal beak [11,19]. The ANs are defined as the aeration of the protuberance on the lateral nasal wall, slightly anterior to the middle turbinate attachment (Figure 4). The Haller cell is an ethmoidal air cell, located below the orbital floor and lateral to a line parallel with the lamina papyracea (Figure 5). Onodi cell is the posteriorly located ethmoidal cell which develops just above and lateral to the sphenoid sinus (Figure 6). Concha bullosa is the pneumatization of the vertical segment of the middle turbinate (Figure 7) [19]. All anatomical variants were initially assessed by a single radiologist with four years of experience in maxillofacial CT imaging, who was blinded to patients’ demographic data, including age and sex. All assessments were done on a DICOM viewer (Medixant. RadiAnt DICOM Viewer [Software]. Version 2024.1. URL: https://www.radiantviewer.com (accessed on 1 November 2024)), after retrieval from the hospital PACS system to provide anonymized evaluation. The prevalence of each variant and the frequency of its bilaterality in each group were recorded. The evaluations were subsequently reviewed by a second radiologist with 11 years of experience. In cases of uncertainty regarding the presence of anatomical variations, a consensus decision was reached in consultation with a third radiologist with 13 years of experience.

### 2.3. Statistical Analysis

A priori power analysis indicated that, assuming a correlation coefficient of r = 0.8 and a 5% significance level (α = 0.05), at least 80–115 subjects were required to achieve 80% power for paired right–left sinus volume comparisons. Since the present study included over 250 participants, the sample size was considered sufficient to ensure adequate statistical power.

The mean volume ± standard deviation (SD) was calculated for each PNS, side, sex and age group. Normal distribution of data was assessed using the Shapiro–Wilk test. The mean ± standard deviation for each measurement parameter was determined independently. The Mann–Whitney U test was used to assess the significance of the differences between males and females for each morphometric parameter. The paired *t*-test was applied to compare each subject’s right and left PNS; an independent samples *t*-test was performed to compare the results of each measurement parameter for the different age groups, interpreting a value of *p* ≤ 0.05 as statistically significant. The data were analyzed using SPSS version 29 (IBM, Armonk, NY, USA).

## 3. Results

### 3.1. Paranasal Sinus Pneumatization and Volume

Sphenoid sinus pneumatization was observed in 30% (3 female, 3 male) of the subjects in Group 1 (0–6 months) (1 only right, 1 only left, 4 bilateral); 75% (9 female, 6 male) in Group 2 (6–12 months) (15 bilateral); 75% (8 female, 7 male) in Group 3 (12–18 months) (15 bilateral); and more than 80% in the older age groups. In subjects between 2 and 8 years old, bilateral absence of sphenoid sinus pneumatization was observed in three cases (2 female, 1 male; 3%), only on the right side in two cases (1 female, 1 male; 2%), and only on the left side in three cases (3 female; 3%). All subjects older than 8 years had bilaterally pneumatized sphenoid sinuses.

The earliest evidence of frontal sinus pneumatization was observed in one male subject (right frontal sinus) in Group 5 (2–3 years). It was present in 30% (4 female, 2 male) of the subjects in Group 6 (3–4 years) (2 only left, 4 bilateral); 65% (7 female, 6 male) in Group 7 (4–5 years) (2 only right, 3 only left, 8 bilateral); 60% (5 female, 7 male) in Group 8 (5–6 years) (1 only right, 2 only left, 9 bilateral); and in more than 90% of the subjects older than 6 years. In subjects over 6 years, bilateral absence of frontal sinus pneumatization was observed in four cases (1 female, 3 male; 4%), only right in one girl (1%), and only left in one boy (1%). All subjects had bilaterally pneumatized maxillary sinuses.

**Figure 8:**
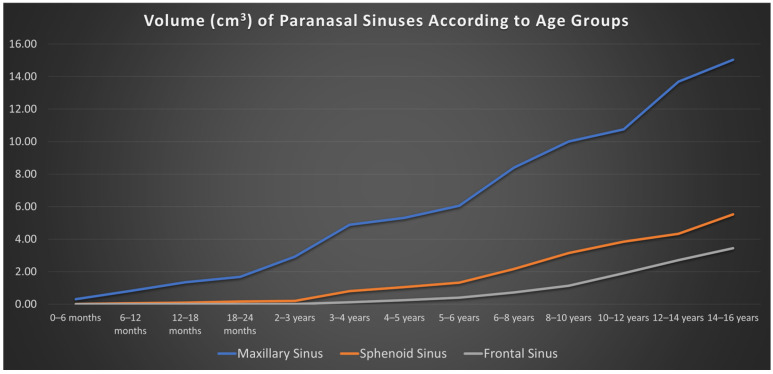
Line graph of the paranasal sinus volumes according to age groups.

The mean volumes ± standard deviation (SD) of the right and left PNS according to age groups in male, female, and total cohorts are presented in Table 1 and Table 2. Overall, no significant differences were found between males and females in terms of PNS volumes (*p* > 0.05). In the entire study group, no significant differences were observed between the right (6.23 cm^3^) and left (6.27 cm^3^) maxillary sinus volumes (*p* = 0.551), nor between the right (0.79 cm^3^) and left (0.86 cm^3^) frontal sinuses (*p* = 0.170). However, a significant difference was found between the right (1.64 cm^3^) and left (1.85 cm^3^) sphenoid sinuses (*p* = 0.041). Comparison of maxillary, sphenoid, and frontal sinus volume growth across age groups is illustrated in Figure 8. During the growth of the sinonasal cavities, the highest Pearson correlation coefficient between right and left sides was obtained in the maxillary sinus (r = 0.980; *p* < 0.001), followed by the frontal sinus (r = 0.840; *p* < 0.001) and sphenoid sinus (r = 0.737; *p* < 0.001).

### 3.2. Sinonasal Variations

The most common were the ANs, observed in 153 cases (58.8%; 117 right, 118 left). Haller cells were found in 81 cases (31.2%; 59 right, 65 left), Onodi cells in 26 cases (10%; 17 right, 19 left), and concha bullosa in 61 cases (23.5%; 46 right, 50 left). Onodi cells emerged after the age of five in our study population, whereas other variations were identified even in infancy. ANs were unilateral in 71 (27.3%) and bilateral in 82 cases, Haller cells were unilateral in 38 (14.6%) and bilateral in 43 cases (16.5%), Onodi cells were unilateral in 14 (16.5%) and bilateral in 12 (4.6%) cases, and concha bullosa cells were unilateral in 26 (7.3%) and bilateral in 35 (13.5%) cases (Table 3). There were no significant differences between boys and girls for any variation [AN (75 male, 78 female; *p* = 0.705), Haller cells (41 male, 40 female; *p* = 0.893), Onodi cells (15 male, 11 female; *p* = 0.408), concha bullosa (30 male, 31 female; *p* = 0.884)].

### 3.3. Correlation Between Sinus Development and Variations

We evaluated whether the presence of sinonasal anatomical variations influenced the volumes of adjacent ipsilateral PNS whose outflow tracts were located nearby. Specifically, right and left ANs were compared with the corresponding frontal sinus volumes, Haller cells with maxillary sinus volumes, Onodi cells with sphenoid sinus volumes, and concha bullosa with all PNS volumes on the same side.

Across all age groups, the mean right frontal sinus volume was 0.94 ± 1.48 cm^3^ in cases with ANs (n = 117) and 0.66 ± 1.35 cm^3^ in those without (n = 143; *p* = 0.118). On the left, mean volumes were 0.89 ± 1.38 cm^3^ with ANs (n = 118) and 0.83 ± 1.44 cm^3^ without (n = 142; *p* = 0.713).

Since Haller cells reach a stable prevalence after approximately three years of age, the comparison of maxillary sinus volumes was performed in patients older than three years. In this subgroup, the mean right maxillary sinus volume was 9.45 ± 3.93 cm^3^ in cases with Haller cells (n = 44) and 9.17 ± 4.37 cm^3^ in those without (n = 116; *p* = 0.704). On the left, the corresponding values were 9.28 ± 3.98 cm^3^ (n = 53) and 9.29 ± 4.68 cm^3^ (n = 107; *p* = 0.582).

Similarly, since Onodi cells are typically observed after the age of five and their frequency stabilizes in late childhood, the comparison of sphenoid sinus volumes was performed in patients older than eight years. In this group, the mean right sphenoid sinus volume was 4.47 ± 2.53 cm^3^ in cases with Onodi cells (n = 17) and 3.79 ± 2.18 cm^3^ in those without (n = 63; *p* = 0.268). On the left, the mean sphenoid sinus volume was 5.01 ± 2.02 cm^3^ with Onodi cells (n = 19) and 4.32 ± 2.15 cm^3^ without (n = 61; *p* = 0.223).

The effect of concha bullosa on ipsilateral PNS volumes was analyzed in patients older than six years. On the right, mean maxillary, sphenoid, and frontal sinus volumes were 11.39 ± 4.19 cm^3^, 4.15 ± 2.54 cm^3^, and 2.06 ± 2.02 cm^3^, respectively, in cases with concha bullosa (n = 31), and 11.62 ± 3.34 cm^3^, 3.36 ± 2.14 cm^3^, and 1.88 ± 1.62 cm^3^ in those without (n = 69; *p* = 0.774, 0.109, and 0.632, respectively). On the left, the mean maxillary, sphenoid, and frontal sinus volumes were 11.13 ± 4.74 cm^3^, 3.54 ± 2.02 cm^3^, and 2.21 ± 1.87 cm^3^ with concha bullosa (n = 32), and 11.83 ± 3.56 cm^3^, 4.21 ± 2.33 cm^3^, and 1.95 ± 1.56 cm^3^ without (n = 68; *p* = 0.459, 0.164, and 0.475, respectively).

## 4. Discussion

Our study provides comprehensive data on PNS growth in pediatric populations with 3D measurements, highlighting that there are no significant differences in sinus volumes or anatomic variants between sexes. In our cohort, the mean volume of the left sphenoid sinus is slightly larger than the right, while no significant difference was found between right and left sides for maxillary and frontal sinuses. The highest correlation of PNS volume with growing age is seen in the maxillary sinus, followed by the frontal and sphenoid sinuses, respectively. In light of these data, it can be concluded that the most reliable volume information reflecting the chronological growth of the facial bones and sinonasal cavity can be obtained from the maxillary sinus.

To evaluate the effect of anatomic variations on normal sinus development—which was one of the main objectives of this study—we compared the sinus volumes on the same side in cases with and without variant cells within each age group. No significant differences were observed in any age group (*p* > 0.05). Based on these findings, we can confidently conclude that the presence of anatomic variants such as ANs, Haller or Onodi cells, or concha bullosa, does not require a different interpretation when assessing sinus volumes according to age.

Consistent with previous studies, the most frequent anatomic variant in our study group was ANs (58.8%), which are known to be present at birth and were observed with similar frequency across all age groups [7,8]. Although the reported prevalence of ANs in the literature varies widely, ranging from 3% to 100%, it is often listed among the three most common sinonasal variants [7,8,20,21]. Pneumatization of these cells has been hypothesized to narrow the frontal recess, potentially impairing the drainage of the frontal sinus and contributing to the development of frontal sinusitis. However, previous studies have found no significant difference in the prevalence of ANs between patients with and without clinically diagnosed frontal rhinosinusitis [7,22]. Similarly, in our study, no significant difference was observed in sinus volumes between individuals with and without ANs, suggesting that their presence does not exert a substantial impact on frontal sinus pneumatization. Nevertheless, in an adult volumetric study, AN volume was reported to show a negative correlation with increasing age, indicating that age-related change should be considered during sinus surgery planning [23].

Haller cells were more frequently identified after the first year of life, whereas Onodi cells tended to appear after the age of five, though their prevalence remained lower than that of other ethmoidal cell variants in older age groups. Similarly to sinus volumes, no significant difference in the prevalence of these anatomic variations was found between male and female patients. Awareness and preoperative identification of Haller and Onodi cells are crucial for surgical planning, as their presence may increase the risk of intraoperative complications [24]. Haller cells, located near the maxillary sinus ostium or the hiatus semilunaris, are associated with a higher risk of orbital injury during ethmoidectomy, while Onodi cells—also known as sphenoethmoidal cells—are situated close to the sphenoid sinus recess and have been linked to optic nerve or internal carotid artery injury during functional endoscopic sinus surgery (FESS) or transsphenoidal procedures [7,24]. Interestingly, the emergence of Onodi cells in parallel with sphenoid sinus pneumatization suggests that, although traditionally classified as posterior ethmoidal cells, they may in fact originate from the sphenoid sinus itself. Despite their anatomical proximity to the drainage pathways of the maxillary and sphenoid sinuses, respectively, our findings demonstrated that the presence of Haller cells did not significantly influence maxillary sinus volume, nor did Onodi cells affect sphenoid sinus volume in the pediatric population.

Previous studies have reported that pneumatization of the middle turbinate begins around the age of five, with its prevalence increasing with age. In a study by Cohen et al., concha bullosa was observed in approximately 10% of children under five years of age. In our cohort, concha bullosa was first detected in 20% of subjects aged 6–12 months, and in 17.5% of subjects aged 6 months to 5 years [11]. Publications prior to 2000 reported concha bullosa prevalence in the second decade as 10–24%, whereas more recent studies have shown a prevalence of 37.5–58% [8,11,20,21]. In our study, the prevalence of concha bullosa in children older than 10 years was 41.6%, consistent with more recent reports. The higher prevalence reported in the recent studies may be attributed to improvements in CT technology, including higher image quality, thinner slice thickness, and increased detector numbers, allowing better detection of small pneumatization. In studies performed on adult populations, concha bullosa prevalence was reported between 35–58%, similar to the findings in our cohort and previous papers of children over 10 years [7,10,11,21]. Based on these findings, it can be inferred that the presence of concha bullosa is generally established by the end of the second decade of life.

The paranasal sinuses originate from the cartilaginous nasal capsule and demonstrate highly individualized and heterogeneous development patterns throughout growth [14]. Despite extensive investigation, the mechanisms regarding paranasal sinus growth remain incompletely elucidated. Several hypotheses have been proposed, including the influence of nasal airflow, cerebral growth, muscular traction forces, facial development, eruption of permanent dentition, and cellular processes such as molecular adhesion and migration [14]. Among these, positive air pressure transmitted from the nasopharynx to the paranasal sinuses is considered a key factor in normal sinus growth; therefore, any obstruction within the nasal–respiratory complex may adversely affect physiologic development.

Functional endoscopic sinus surgery (FESS) is increasingly utilized in pediatric patients with recurrent or chronic rhinosinusitis refractory to maximal medical therapy. However, previous studies have reported alterations in facial growth following various surgical interventions performed during childhood, including FESS, nasal septoplasty, midfacial fracture repair, early cleft palate repair, and the Caldwell–Luc procedure [14,25]. Similarly, in adult populations, reduced maxillary sinus pneumatization has been demonstrated on the side of increased tooth loss, suggesting a close interaction between dentition and sinus development [25,26]. In this context, for children undergoing paranasal sinus or dental surgery during growth, longitudinal follow-up incorporating population-specific, age-adjusted normative paranasal sinus charts that reflect the patient’s ethnic background may provide valuable reference standards. Such an approach could facilitate the detection of subtle facial asymmetries and enable more accurate predictions of future growth patterns, thereby enhancing clinical assessment and long-term patient management.

Numerous studies in the literature have evaluated the volume of PNS in different age groups from various countries. Ten pediatric studies with comparable methodology are summarized in Table S1. When comparing our results with a study conducted in New Zealand, which used the same software and a similar segmentation-based methodology but included broader age intervals and fewer subgroups, maxillary sinus volumes in our cohort were found to be higher in subjects younger than 4 years, whereas slightly lower in those older than 4 years [27]. In contrast, comparison with a South African study using the same volumetric calculation system for all PNS showed that our cohort had higher maxillary, sphenoid, and total sinus volumes across all age groups, while frontal sinus volumes were comparable between two populations [28]. Similarly, when compared with a study conducted in a Polish population in which maxillary sinus volumes were stratified by age, our results consistently demonstrated slightly higher volumes across all age groups [18]. In line with this observation, Gruszka et al. reported that the incidental prevalence of frontal and maxillary sinus hypoplasia in the adult Turkish Cypriot population was significantly lower than that observed in a Polish population [21].

The sharp increase in sinus volume between the 3–4-year age range reported by Lee et al. and Adibelli et al. was not observed in our study or most others, where a steady growth trend was maintained [27,29]. This discrepancy likely reflects the heterogeneity of age interval classifications among studies and the lack of access to individual age data within subgroups. When compared to Adibelli et al. [29], our cohort showed notably smaller maxillary sinus volumes in subjects older than 6 years, similar sphenoid sinus volumes across all age groups, and slightly smaller frontal sinus volumes after 12 years. However, another Turkish study found the opposite pattern, with smaller maxillary sinus volumes than our cohort in participants older than 6 years, while a third study from Turkey reported similar PNS volumes to ours [29,30]. Despite sharing the same ethnicity, the discrepancies among these three Turkish studies and our findings are most likely due to methodological differences. In our study, sinus volumes were calculated by manual segmentation of each CT slice, whereas the other studies estimated volumes indirectly using geometric formulas derived from two-dimensional length or area measurements [15,29,30]. Given that maxillary and frontal sinuses do not conform well to ellipsoid geometry, this may explain the variation in reported volumes. Notably, among the three Turkish studies using similar protocols, one yielded lower, one higher, and one comparable result to ours, supporting the higher reliability and interobserver consistency of true 3D volumetric segmentation [15,29,30]. Additionally, the use of MRI instead of CT in one study may have contributed to further measurement discrepancies due to less precise delineation of sinus walls [29].

Comparing with a Japanese study, maxillary sinus volumes in our cohort were smaller in subjects older than 10 years, whereas sphenoid and frontal sinus volumes were similar between the two populations [12]. In the South Korean cohort, maxillary sinus volumes were higher than ours between 3–9 and 13–16 years, while sphenoid sinus volumes were consistently smaller, and frontal sinus volumes were smaller until 9 years of age [16]. Among studies with wider age ranges, subjects in the Mexican study demonstrated substantially larger PNS volumes compared to our results and others, whereas Swiss and Polish studies reported smaller maxillary sinus volumes—particularly under 8 years—with similar sphenoid sinus volumes [4,17,18]. Considering ethnic differences, geographic latitude and climate, these findings may suggest that PNS development in early childhood may progress more rapidly in regions with warmer climates and greater sunlight exposure.

Regarding sex differences, although some reports identified slight variations in specific subgroups, most found no statistically significant differences in mean sinus volumes between males and females—consistent with our findings [2,25,31]. Therefore, we suggest that normative reference charts for pediatric PNS volumes can be presented without stratification by sex.

In side-to-side comparison, only the left sphenoid sinus was significantly larger than the right in our cohort. However, since most previous studies reported no significant right–left asymmetry and some of our subgroups also lacked side-specific differences, we consider this finding specific to our sample rather than generalizable to the broader population. As with sex-based analysis, we recommend providing reference values without right/left distinction for practical use in routine reporting.

The main limitation of our study is the classification according to age and sex, not including height, weight, growth percentile, or head circumference, which may impact sinus size. Second, relatively small sample size for each age group, which may affect the stability of *p*-values and increase the risk of false-positive or false-negative results. Third, we did not evaluate the effect of pathologic conditions such as allergic conditions, asthma, or drug use on PNS growth, as we only evaluated the cases without any sinonasal mucosal disease. Fourth, other important anatomical variations such as Kuhn cells, olfactory fossa depth (Keros classification), nasal septal deviation, and paradoxical middle turbinate were not assessed in this study, as our analysis focused on those variants that were considered most likely to influence sinus development. Fifth, intra- or interobserver agreement analyses were not performed since sinus volumes were determined by manual segmentation, which inherently minimizes measurement discrepancies when performed by the single experienced observer. Likewise, previous studies using the same volumetric calculation method have demonstrated an excellent level of consistency (ICC > 0.9) [23,26,27]. Therefore, instead of performing a separate interobserver analysis, two radiologists jointly performed the manual segmentations in consensus. For the identification of anatomical variations, all evaluations were performed in consensus by two radiologists, ensuring consistency and diagnostic accuracy. Lastly, ethnic differences—which have been reported to influence PNS morphology and volume in previous studies—were not considered in our analysis. However, all participants in our cohort were of Middle Eastern or Caucasian descent, including Turkish, Kurdish, and Syrian individuals, which provides relative ethnic homogeneity within the study population. Strengths of our study include a large study group containing balanced subgroups with equally distributed boys and girls. Also, volumetric evaluation was performed with segmentation from multiple slides.

## 5. Conclusions

By combining consecutive age groups with no statistically significant differences, we established broader and more practical age intervals separately for each sinus—six for the maxillary sinus, five for the frontal sinus, and four for the sphenoid sinus (Table 4). This approach allowed us to create a simplified, clinically applicable reference chart that reflects the natural growth pattern of each sinus. The chart provides radiologists and clinicians with an opportunity to compare a child’s sinus volumes with age-specific reference ranges, facilitating the evaluation of developmental delay, craniofacial anomalies (such as Apert or Crouzon syndromes), or chronic PNS diseases in relation to expected growth. Future studies with larger multicenter cohorts and inclusion of parameters such as body mass index, craniofacial index, and environmental exposure would provide a more comprehensive understanding of factors affecting sinus development. Additionally, integration of automated segmentation tools and artificial intelligence-based volumetric analysis may further improve the precision and reproducibility of sinus volume assessment in pediatric populations.

## Figures and Tables

**Table 1:** Mean volume and standard deviations (SD) of paranasal sinuses of boys and girls according to age groups.

Age Groups	Maxillary Sinus			Sphenoid Sinus			Frontal Sinus		
	♂ (Mean ± SD) (cm^3^)	♀ (Mean ± SD) (cm^3^)	Min–Max (cm^3^)	♂ (Mean ± SD) (cm^3^)	♀ (Mean ± SD) (cm^3^)	Min–Max (cm^3^)	♂ (Mean ± SD) (cm^3^)	♀ (Mean ± SD) (cm^3^)	Min–Max (cm^3^)
0–6 months	0.30 ± 0.09	0.33 ± 0.15	0.13–0.60	0.01 ± 0.03	0.01 ± 0.03	0–0.11	-	-	-
6–12 months	0.95 ± 0.30	0.67 ± 0.38	0.27–1.72	0.08 ± 0.08	0.05 ± 0.04	0–0.25	-	-	-
12–18 months	1.33 ± 0.41	1.37 ± 0.34	0.5–2.06	0.09 ± 0.08	0.10 ± 0.10	0–0.31	-	-	-
18–23.99 months	1.72 ± 0.44	1.64 ± 0.33	1.17–2.72	0.19 ± 0.18	0.16 ± 0.15	0–0.61	-	-	-
2–3 years	3.20 ± 0.82	2.63 ± 0.77	1.67–4.84	0.22 ± 0.14	0.19 ± 0.11	0–0.52	0.01 ± 0.05	-	0–0.22
3–4 years	5.54 ± 1.60	4.25 ± 1.22	2.61–9.09	0.79 ± 0.73	0.80 ± 0.76	0–2.7	0.16 ± 0.32	0.09 ± 0.13	0–1.4
4–5 years	5.36 ± 1.75	5.27 ± 1.48	2.27–8.26	1.14 ± 0.81	0.95 ± 0.69	0–3.07	0.21 ± 0.25	0.28 ± 0.35	0–1.14
5–6 years	5.90 ± 1.77	6.19 ± 1.49	3.12–9.98	1.13 ± 0.75	1.52 ± 0.95	0–4.49	0.37 ± 0.49	0.41 ± 0.49	0–2.11
6–8 years	8.95 ± 2.32	7.83 ± 1.82	4.27–15.78	2.24 ± 1.25	2.07 ± 2.04	0–8.27	0.81 ± 0.67	0.63 ± 0.58	0–2.45
8–10 years	10.48 ± 0.99	9.53 ± 1.73	6.44–13.57	2.66 ± 1.23	3.61 ± 1.65	0.32–6.34	1.21 ± 1.04	1.06 ± 0.53	0–4.2
10–12 years	11.16 ± 2.85	10.34 ± 3.21	1.96–17.37	4.08 ± 1.66	3.59 ± 1.29	0.41–10.48	1.78 ± 1.32	2.02 ± 1.29	0–5.62
12–14 years	12.92 ± 2.21	14.46 ± 3.09	10.17–20.37	4.41 ± 1.39	4.25 ± 1.31	0.59–7.24	2.99 ± 1.79	2.43 ± 1.26	0–6.7
14–16 years	15.52 ± 5.31	14.52 ± 2.85	5.55–23.53	6.13 ± 1.65	4.91 ± 1.56	2.01–10.5	4.36 ± 1.63	2.51 ± 1.08	0–8.37

**Table 2:** Mean volume and standard deviations (SD) of right and left paranasal sinuses according to age groups.

Age Groups	Maxillary Sinus				Sphenoid Sinus				Frontal Sinus			
	Right (Mean ± SD) (cm^3^)	Right (Min–Max) (cm^3^)	Left (Mean ± SD) (cm^3^)	Left (Min–Max) (cm^3^)	Right (Mean ± SD) (cm^3^)	Right (Min–Max) (cm^3^)	Left (Mean ± SD) (cm^3^)	Left (Min–Max) (cm^3^)	Right (Mean ± SD) (cm^3^)	Right (Min–Max) (cm^3^)	Left (Mean ± SD) (cm^3^)	Left (Min–Max) (cm^3^)
0–5.99 months	0.31 ± 0.13	0.13–0.60	0.32 ± 0.14	0.13–0.55	0.01 ± 0.02	0–0.06	0.01 ± 0.02	0–0.11	-	-	-	-
6–11.99 months	0.82 ± 0.42	0.27–1.72	0.79 ± 0.32	0.31–1.46	0.06 ± 0.06	0–0.16	0.07 ± 0.07	0–0.25	-	-	-	-
12–17.99 months	1.33 ± 0.36	0.55–2.01	1.37 ± 0.39	0.5–2.06	0.1 ± 0.1	0–0.31	0.09 ± 0.09	0–0.29	-	-	-	-
18–23.99 months	1.62 ± 0.35	1.17–2.42	1.75 ± 0.47	0.94–2.72	0.18 ± 0.18	0–0.58	0.17 ± 0.15	0–0.61	-	-	-	-
2–3 years	2.92 ± 0.92	1.67–4.84	2.91 ± 0.79	1.72–4.62	0.21 ± 0.14	0.03–0.52	0.19 ± 0.13	0–0.38	0.01 ± 0.05	0–0.22	-	-
3–4 years	5.03 ± 1.61	2.88–9.09	4.76 ± 1.54	2.61–8.75	0.73 ± 0.72	0–2.29	0.87 ± 0.78	0–2.7	0.06 ± 0.13	0–0.49	0.19 ± 0.39	0–1.4
4–5 years	5.23 ± 1.60	2.30–8.07	5.39 ± 1.63	2.27–8.26	0.92 ± 0.77	0–3.07	1.17 ± 0.94	0.04–3.07	0.22 ± 0.31	0–1.09	0.28 ± 0.35	0–1.14
5–6 years	5.96 ± 1.63	3.12–9.98	6.14 ± 1.65	3.57–9.76	1.15 ± 0.83	0–2.56	1.51 ± 1.08	0–4.49	0.29 ± 0.41	0–1.22	0.50 ± 0.64	0–2.11
6–8 years	8.53 ± 2.31	5.07–15.78	8.26 ± 2.07	4.27–14.12	2.29 ± 1.96	0–8.27	2.02 ± 1.53	0–5.94	0.63 ± 0.69	0–2.45	0.81 ± 0.71	0–2.37
8–10 years	10.07 ± 1.63	6.44–13.57	9.95 ± 1.47	7.56–13.4	2.89 ± 1.95	0.32–6.34	3.39 ± 1.75	0.5–5.96	1.09 ± 0.84	0–2.99	1.18 ± 0.99	0–4.2
10–12 years	10.87 ± 2.80	6.51–17.37	10.63 ± 3.36	1.96–16.77	3.18 ± 1.81	1.04–6.82	4.50 ± 2.27	0.41–10.48	1.48 ± 1.26	0–4.46	2.32 ± 1.63	0–5.62
12–14 years	13.53 ± 2.67	10.17–18.96	13.85 ± 3.00	10.19–20.37	4.03 ± 1.46	0.59–6.64	4.64 ± 2.01	1.35–7.24	2.76 ± 1.52	0.46–6.7	2.67 ± 1.74	0–6.7
14–16 years	14.74 ± 4.18	7.23–22.13	15.32 ± 4.43	5.55–23.53	5.63 ± 2.68	2.01–9.88	5.42 ± 2.10	2.81–10.5	3.70 ± 2.01	1.01–8.37	3.18 ± 1.71	0–6.73

**Table 3:** Prevalence of sinonasal anatomic variants according to age group and sex.

Age	Agger Nasi Cell		Haller Cell		Onodi Cell		Concha Bullosa	
Groups	Unilateral-R/L, (%) Bilateral n (%)		Unilateral-R/L (%) Bilateral n (%)		Unilateral-R/L (%) Bilateral n (%)		Unilateral-R/L (%) Bilateral n(%)	
0–5.99 months	2/4 (30%)	5 (25%)	0/0	1 (5%)	0/0	0	0/0	0
6–11.99 months	0/4 (20%)	6 (30%)	1/2 (15%)	0	0/0	0	2/0 (10%)	2 (10%)
12–17.99 months	4/4 (40%)	5 (25%)	1/1 (10%)	1 (5%)	0/0	0	0/2 (10%)	0
18–23.99 months	3/1 (20%)	6 (30%)	1/2 (15%)	3 (15%)	0/0	0	1/1 (10%)	1 (5%)
2–3 years	3/4 (35%)	3 (15%)	5/0 (25%)	2 (10%)	0/0	0	0/0	0
3–4 years	3/2 (25%)	8 (40%)	1/4 (25%)	4 (20%)	0/0	0	2/4 (30%)	1 (5%)
4–5 years	4/3 (35%)	4 (20%)	1/2 (15%)	5 (25%)	0/0	0	0/1 (5%)	4 (20%)
5–6 years	1/2 (15%)	10 (50%)	1/1 (10%)	3 (15%)	0/1 (5%)	0	0/0	2 (10%)
6–8 years	4/3 (35%)	6 (30%)	1/3 (20%)	5 (25%)	0/1 (5%)	0	1/2 (15%)	5 (25%)
8–10 years	2/2 (20%)	6 (30%)	2/0 (10%)	8 (40%)	1/2 (15%)	2 (10%)	0/1 (5%)	4 (20%)
10–12 years	3/1 (20%)	8 (40%)	1/2 (15%)	3 (15%)	1/0 (5%)	2 (10%)	1/0 (5%)	8 (40%)
12–14 years	0/4 (20%)	8 (40%)	0/4 (20%)	5 (25%)	1/2 (15%)	5 (20%)	2/4 (30%)	4 (20%)
14–16 years	6/2 (40%)	7 (35%)	1/1 (10%)	3 (15%)	2/3 (20%)	3 (15%)	2/0 (10%)	4 (20%)
Total	35/36 (27.3%)	82 (31.5%)	16/22 (14.6%)	43 (16.5%)	5/9 (5.4%)	12 (4.6%)	11/15 (10%)	35 (13.5%)

**Table 4:** Practical chart of normal range of paranasal sinus volumes according to age groups in pediatric population.

Age Range	Maxillary Sinus [Mean (Min–Max)] (cm^3^)	Sphenoid Sinus [Mean (Min–Max)] (cm^3^)	Frontal Sinus [Mean (Min–Max)] (cm^3^)
0–18 months	0.82 (<1.7)	0.11 (<0.4)	0.10 (<1.4)
18–36 months	2.29 (1.3–3.7)		
3–6 years	5.42 (3.3–8)	1.05 (0.1–2.2)	
6–9 years	9.20 (5.5–11.5)	2.64 (0.6–4.7)	0.92 (0.1–2.5)
9–10 years	10.38 (8.5–14)		
10–12 years		4.56 (2.4–7.2)	1.90 (0.7–4.1)
12–14 years	14.35 (11–20)		2.71 (1.2–5.5)
14–16 years			3.44 (1.8–5.9)

## Data Availability

The datasets generated and/or analyzed during the current study are available from the corresponding author upon reasonable request.

## Supplementary Materials

The following supporting information can be downloaded at: https://www.mdpi.com/article/10.3390/tomography12020015/s1, Table S1: Previous 10 articles in the literature evaluating the paranasal sinus volumes with similar pediatric age group and methodology.

## Abbreviations

The following abbreviations are used in this manuscript:

PACS	Picture Archiving and Communication System
SD	Standard Deviation
PNS	Paranasal Sinus
ANs	Agger Nasi cells

## References

[1] Iturralde-Garrote A., Sanz J.L., Forner L., Melo M., Puig-Herreros C. (2023). Volumetric Changes of the Paranasal Sinuses with Age: A Systematic Review. J. Clin. Med..

[2] Oliveira J.M., Alonso M.B., de Sousa E., Tucunduva M.J., Fuziy A., Scocate A.C., Costa A.L. (2017). Volumetric study of sphenoid sinuses: Anatomical analysis in helical computed tomography. Surg. Radiol. Anat..

[3] Tatlisumak E., Ovali G.Y., Asirdizer M., Aslan A., Ozyurt B., Bayindir P., Tarhan S. (2008). CT study on morphometry of frontal sinus. Clin. Anat..

[4] Jasso-Ramírez N.G., Elizondo-Omaña R.E., Treviño-González J.L., Quiroga-Garza A., Garza-Rico I.A., Aguilar-Morales K., Elizondo-Riojas G., Guzmán-Lopez S. (2023). Morphometric variants of the paranasal sinuses in a Mexican population: Expected changes according to age and gender. Folia Morphol..

[5] Alkire B.C., Bhattacharyya N. (2010). An assessment of sinonasal anatomic variants potentially associated with recurrent acute rhinosinusitis. Laryngoscope.

[6] Fadda G.L., Rosso S., Aversa S., Petrelli A., Ondolo C., Succo G. (2012). Multiparametric statistical correlations between paranasal sinus anatomic variations and chronic rhinosinusitis. Acta Otorhinolaryngol. Ital..

[7] Shpilberg K.A., Daniel S.C., Doshi A.H., Lawson W., Som P.M. (2015). CT of Anatomic Variants of the Paranasal Sinuses and Nasal Cavity: Poor Correlation With Radiologically Significant Rhinosinusitis but Importance in Surgical Planning. AJR Am. J. Roentgenol..

[8] Al-Qudah M. (2008). The relationship between anatomical variations of the sino-nasal region and chronic sinusitis extension in children. Int. J. Pediatr. Otorhinolaryngol..

[9] Comer B.T., Kincaid N.W., Smith N.J., Wallace J.H., Kountakis S.E. (2013). Frontal sinus septations predict the presence of supraorbital ethmoid cells. Laryngoscope.

[10] Smith K.D., Edwards P.C., Saini T.S., Norton N.S. (2010). The prevalence of concha bullosa and nasal septal deviation and their relationship to maxillary sinusitis by volumetric tomography. Int. J. Dent..

[11] Cohen O., Adi M., Shapira-Galitz Y., Halperin D., Warman M. (2019). Anatomic variations of the paranasal sinuses in the general pediatric population. Rhinology.

[12] Yamakawa K., Nishijima H., Koizumi M., Kondo K. (2024). Assessing volume growth of paranasal sinuses and nasal cavity in children using three-dimensional imaging software. Auris Nasus Larynx.

[13] von Elm E., Altman D.G., Egger M., Pocock S.J., Gøtzsche P.C., Vandenbroucke J.P., STROBE Initiative (2008). The Strengthening the Reporting of Observational Studies in Epidemiology (STROBE) statement: Guidelines for reporting observational studies. J. Clin. Epidemiol..

[14] Lee S., Fernandez J., Mirjalili S.A., Kirkpatrick J. (2022). Pediatric paranasal sinuses-Development, growth, pathology, & functional endoscopic sinus surgery. Clin. Anat..

[15] Değermenci M., Ertekin T., Ülger H., Acer N., Coşkun A. (2016). The Age-Related Development of Maxillary Sinus in Children. J. Craniofac Surg..

[16] Park I.H., Song J.S., Choi H., Kim T.H., Hoon S., Lee S.H., Lee H.M. (2010). Volumetric study in the development of paranasal sinuses by CT imaging in Asian: A pilot study. Int. J. Pediatr. Otorhinolaryngol..

[17] Barghouth G., Prior J.O., Lepori D., Duvoisin B., Schnyder P., Gudinchet F. (2002). Paranasal sinuses in children: Size evaluation of maxillary, sphenoid, and frontal sinuses by magnetic resonance imaging and proposal of volume index percentile curves. Eur. Radiol..

[18] Lorkiewicz-Muszyńska D., Kociemba W., Rewekant A., Sroka A., Jończyk-Potoczna K., Patelska-Banaszewska M., Przystańska A. (2015). Development of the maxillary sinus from birth to age 18. Postnatal growth pattern. Int. J. Pediatr. Otorhinolaryngol..

[19] Lund V.J., Stammberger H., Fokkens W.J., Beale T., Brenal-Spreksen M., Eloy P., Georgalas C., Gerstenberger C., Hellings P., Herman P. (2014). European position paper on the anatomical terminology of the internal nose and paranasal sinuses-a free resource. Rhinol. Suppl..

[20] Kim H.J., Jung Cho M., Lee J.W., Tae Kim Y., Kahng H., Sung Kim H., Hahm K.H. (2006). The relationship between anatomic variations of paranasal sinuses and chronic sinusitis in children. Acta Otolaryngol..

[21] Gruszka K., Aksoy S., Rozylo-Kalinowska I., Gülbeş M.M., Kalinowski P., Orhan K. (2022). A comparative study of paranasal sinus and nasal cavity anatomic variations between the Polish and Turkish Cypriot Population with CBCT. Head. Face Med..

[22] Eweiss A.Z., Khalil H.S. (2013). The prevalence of frontal cells and their relation to frontal sinusitis: A radiological study of the frontal recess area. ISRN Otolaryngol..

[23] Ricardo A.L.F., Ogawa C.M., Gomes J.P.P., De Rosa C.S., Lopes S.L.P.d.C., Braz-Silva P.H., Orhan K., Costa A.L.F. (2022). Three-Dimensional Volumetric Analysis of Frontal Ethmoidal Cells and Evaluation of Influential Factors: A Helical Computed Tomography Study. Tomography.

[24] Nouraei S.A., Elisay A.R., Dimarco A., Abdi R., Majidi H., Madani S.A., Andrews P.J. (2009). Variations in paranasal sinus anatomy: Implications for the pathophysiology of chronic rhinosinusitis and safety of endoscopic sinus surgery. J. Otolaryngol. Head. Neck Surg..

[25] Borges Tanaka L.E., Franco A., Abib R.F., Manhães-Junior L.R.C., de Castro Lopes S.L.P. (2021). Age- and sex-related changes of the volume of maxillary sinuses quantified via cone beam computed tomography. Res. Soc. Dev..

[26] Ozsoy S.C., Bahrilli S., Altiparmak F., Yilmaz H., Yuksel I.B., Arslan M.E., Altindag A. (2025). Volumetric evaluation of the maxillary sinus using cone-beam computed tomography. BMC Oral Health.

[27] Lee S., Fernandez J.W., Mahadevan M., Tarr G., Mirjalili A. (2020). Using 3D-reconstruction to analyse typical growth trends of the maxillary sinus in children. Int. J. Pediatr. Otorhinolaryngol..

[28] Rennie C.O., Haffajee M.R., Satyapal K.S. (2017). Development of the paranasal air sinuses in a South African population utilising three dimensional (3D) reconstructed models. Eur. J. Anat..

[29] Adibelli Z.H., Songu M., Adibelli H. (2011). Paranasal sinus development in children: A magnetic resonance imaging analysis. Am. J. Rhinol. Allergy.

[30] Karakas S., Kavakli A. (2005). Morphometric examination of the paranasal sinuses and mastoid air cells using computed tomography. Ann. Saudi Med..

[31] Tuang G.J., Zahedi F.D., Husain S., Hamizan A.K.W., Kew T.Y., Thanabalan J. (2023). Volumetric evaluation of the sphenoid sinus among different races in the Southeast Asian (SEA) population: A computerized tomography study. Int. J. Med. Sci..

