# Supplementary material for: Assessment of Paranasal Sinus Growth with 3D Volumetric Measurements and the Effect of Anatomic Variations on Sinus Volume in a Pediatric Population
Source: Tomography. 2026 Jan 26;12(2):15. doi: 10.3390/tomography12020015 (PMC12944150; doi:10.3390/tomography12020015)
Supplement: Supplementary file 1 [file tomography-12-00015-s001.zip › Table S1_revised.pdf]

Table S1. Previous 10 articles in the literature evaluating the paranasal sinus volumes with similar paediatric age group and methodology

Author, Year and Country	Methods Used to Assess Sinus Volume	Total sample size (Male, Female)	Age groups (years) [sample size]	Maxillary (R) mean ± SD or [min-max]	Maxillary (L) mean ± SD or [min-max]	Sphenoid (R) mean ± SD or [min-max]	Sphenoid (L) mean ± SD or [min-max]	Frontal (R) mean ± SD or [min-max]	Frontal (L) mean ± SD or [min-max]
**1. Barghouth et al., 2002, Switzerland[17]**	Volume was mesaured from MRI images by measuring three diameters and following simplified ellipsoid formula was used: width x ant-post.x height x 0.5)	Maxillary sinus: 159 (94,59) Sphenoid sinus: 95 (64,31)	<1	0.08 [0.02–0.14]		0.01 [0–0.01]			
			2	1 [0.4–1.6]		0.1 [0.06–0.17]			
			4	2.7 [1.3–4.1]		0.32 [0.18–0.57]			
			8	6.9 [4–10.1]		0.90 [0.48–1.77]			
			12	11.9 [7.7–17.1]		1.66 [0.86–3.44]			
			16	18.3 [12.8–25.9]		2.69 [1.37–5.82]			
**2. Karakas et al., 2005, Turkiye[30]**	CT slice area was calculated, and by multiplying it by the slice thickness, the volume was calculated. By adding all the partial volumes together, the total volume was calculated according to the Cavalieri Principle	91 (47,44) (including older age groups, not presented in this table)	5–10 [18]	5.34±0.56(♂)<br>7.03±2.02(♀)	6.70 ± 1.10 (♂)<br>6.60 ± 2.25 (♀)	2.96 ± 2.53 (♂)<br>3.14 ± 2.30 (♀)		1.19 ± 0.69 (♂)<br>1.23 ± 0.29 (♀)	
			11–15 [18]	11.34 ± 3.10 (♂)<br>10.03 ± 4.41 (♀)	11.01±2.54(♂)<br>9.57 ± 4.48(♀)	5.40 ± 1.96 (♂)<br>4.85 ± 1.09 (♀)		4.20 ± 3.98 (♂)<br>1.75 ± 1.42 (♀)	
			16–20 [17]	14.74 ± 5.79 (♂)<br>14.29 ± 3.42 (♀)	14.55± 4.72(♂)<br>13.78± 3.41(♀)	7.50 ± 3.21 (♂)<br>5.43 ± 2.59 (♀)		7.57 ± 5.72 (♂)<br>3.54 ± 2.25 (♀)	
**3. Park et al., 2010, South Korea[16]**	The volume of sinuses was automatically calculated in the 3D reconstruction by Software Vworks 4.0	250 (169,91) (including older age groups, not presented in this table)	0–1 [10]	0.745 ± 0.035		no pneumatization		no pneumatization	
			1–2 [10]	2.234 ± 0.284		0.018 ± 0.014		no pneumatization	
			2–3 [10]	2.275 ± 0.120		0.215 ± 0.050		0.003 ± 0.003	
			3–4 [10]	3.165 ± 0.208		0.200 ± 0.146		0.003 ± 0.006	
			4–5 [10]	3.996 ± 0.533		0.311 ± 0.161		0.004 ± 0.010	
			5–6 [10]	4.739 ± 0.489		0.669 ± 0.204		0.012 ± 0.018	
			6–7 [10]	5.893 ± 0.356		0.593 ± 0.209		0.159 ± 0.124	
			7–8 [10]	7.295 ± 0.966		1.867 ± 1.125		0.464 ± 0.121	
			8–9 [10]	6.301 ± 0.450		1.424 ± 0.179		0.700 ± 0.229	
			9–10 [10]	10.127 ± 1.552		3.775 ± 0.821		2.029 ± 0.738	

			10–11 [10]	9.389 ± 0.573	3.869 ± 0.884	1.795 ± 0.328
			11–12 [10]	10.706 ± 0.676	3.361 ± 0.555	1.897 ± 0.700
			12–13 [10]	11.997 ± 1.094	3.602 ± 0.590	2.003 ± 0.755
			13–14 [10]	12.239 ± 1.057	3.583 ± 0.849	2.786 ± 1.106
			14–15 [10]	12.232 ± 0.965	3.749 ± 0.715	2.818 ± 0.649
			15–16 [10]	13.110 ± 1.264	3.932 ± 0.408	3.366 ± 0.853
**4. Adibelli et al., 2011, Turkiye**[29]	Volume was mesaured from MRI images by measuring three diameters and following simplified ellipsoid formula was used: width x ant-post.x height x 0.5)	1452	0–2.9 months [135]	0.16 ± 0.27 [0–1.68]	no pneumatization	no pneumatization
			3–5.9 months [138]	0.45 ± 0.44 [0–2.12]	no pneumatization	no pneumatization
			6–8.9 months [117]	0.64 ± 0.36 [0–1.6]	0.004 ± 0.02 [0–0.14]	no pneumatization
			9–11.9 months [123]	1.34 ± 0.73 [0.74–3.37]	0.03 ± 0.06 [0–0.15]	no pneumatization
			1–2.9 [144]	2.96 ± 2.52 [0.13–10.95]	0.09 ± 0.15 [0–0.95]	no pneumatization
			3–5.9 [144]	6.83 ± 3.41 [2.43–17.98]	0.99 ± 0.99 [0.11–3.62]	0.25 ± 0.45 [0–1.52]
			6–8.9[183]	11.05 ± 3.51 [4.12–16.93]	1.69 ± 0.93 [0.20–4.24]	0.93 ± 0.85 [0–3.15]
			9-11.9[165]	14.39 ± 5.70 [4.82–26.90]	3.30 ± 1.94 [0.20–8.25]	1.64 ± 1.21 [0.43–6.24]
			12-14.9[165]	17.79 ± 6.26 [7.17–30.82]	4.65 ± 2.36 [1.65–11.91]	3.68 ± 2.37 [0.65–10.26]
			15–18 [138]	23.65 ± 9.67 [12.47–39.61]	4.96 ± 1.77 [2.41–8.65]	6.28 ± 5.35 [2.22–17.15]
**5. Lorkiewicz-Muszynska et al., 2015, Poland**[18]	Average of sphere ($V = 4/3\Pi r^3$) and pyramid (1/3 width*length*height) volume formula after measurement of three diameters. The diameter of the sphere was calculated as the mean of three measurements	170 (85,85) Equally distributed in each age group	0–2 [10]	0.10 ± 0.03 (♂) 0.37 ± 0.28(♀)		
			2–3 [10]	1.83 ± 1.15 (♂) 1.41 ± 0.53 (♀)		
			3–4 [10]	2.48 ± 0.84 (♂) 3.05 ± 0.82 (♀)		
			4–5 [10]	3.18 ± 0.67 (♂) 4.17 ± 2.64 (♀)		
			5–6 [10]	4.38 ± 1.23 (♂) 4.88 ± 2.46 (♀)		
			6–7 [10]	5.41 ± 1.14 (♂) 4.87 ± 1.12 (♀)		
			7–8 [10]	6.64 ± 2.76 (♂) 5.02 ± 1.71 (♀)		
			8–9 [10]	8.15 ± 1.56 (♂) 6.08 ± 0.90 (♀)		
			9–10 [10]	8.92 ± 2.07 (♂) 10.09 ±3.28(♀)		
			10–11 [10]	7.83 ± 1.94 (♂) 9.61 ± 2.00 (♀)		
			11–12 [10]	9.84 ± 2.04 (♂) 10.12 ±2.87(♀)		

			12–13 [10]	12.13 ±2.29 (♂)11.19±2.60(♀)		
			13–14 [10]	12.77 ± 2.24(♂)12.94±1.79(♀)		
			14–15 [10]	13.25 ± 2.03(♂)13.97±1.97(♀)		
			15–16 [10]	17.18 ± 1.82(♂)10.84±1.47(♀)		
**6. Degermenci et al., 2016, Turkey** [15]	1. Ellipsoid formula using morphological parameters. 2. Stereological analysis using the Cavalieri principle (pointcounting method).	361 (181,180) Equally distributed in each age group and including older age groups, not presented in this table	0–1 [20]	0.95 ± 1.82 (1) 0.95 ± 1.60 (2)	0.98 ± 1.68 (1) 0.91 ± 1.45 (2)	
			1–2 [20]	1.81 ± 0.92 (1) 1.77 ± 0.85 (2)	1.81 ± 0.88 (1) 1.78 ± 0.76 (2)	
			2–3 [20]	3.26 ± 1.22 (1) 3.20 ± 1.26 (2)	3.32 ± 1.22 (1) 3.28 ± 1.20 (2)	
			3–4 [20]	4.34 ± 1.70 (1) 4.33 ± 1.71 (2)	4.42 ± 2.14 (1) 4.32 ± 1.96 (2)	
			4–5 [20]	5.01 ± 1.46 (1) 4.98 ± 1.49 (2)	4.78 ± 1.50 (1) 4.72 ± 1.56 (2)	
			5–6 [20]	5.29 ± 1.86 (1) 5.22 ± 1.88 (2)	5.51 ± 1.84 (1) 5.46 ± 1.85 (2)	
			6–7 [20]	6.68 ± 1.28 (1) 6.60 ± 1.36 (2)	6.61 ± 1.58 (1) 6.61 ± 1.62 (2)	
			7–8 [20]	7.60 ± 2.71 (1) 7.59 ± 2.75 (2)	7.75 ± 2.56 (1) 7.78 ± 2.77 (2)	
			8–9 [20]	7.52 ± 2.84 (1) 7.46 ± 2.84 (2)	7.79 ± 2.70 (1) 7.77 ± 2.66 (2)	
			9–10 [20]	8.66 ± 2.67 (1) 8.08 ± 3.06 (2)	8.48 ± 2.58 (1) 8.07 ± 3.08 (2)	
			10–11 [20]	9.26 ± 3.11 (1) 9.35 ± 2.89 (2)	9.52 ± 3.38 (1) 9.34 ± 3.19 (2)	
			11–12 [20]	10.11 ± 3.08 (1) 9.87 ± 2.84 (2)	10.19 ± 2.85 (1) 9.96 ± 2.74 (2)	
			12–13 [20]	9.61 ± 3.08 (1) 9.60 ± 3.00 (2)	10.36 ± 2.08 (1) 10.31 ± 2.00 (2)	
			13–14 [21]	11.32 ± 4.85 (1) 11.48 ± 4.78 (2)	11.97 ± 5.08 (1) 11.80 ± 4.84 (2)	
			14–15 [20]	12.70 ± 3.23 (1) 12.56 ± 2.90 (2)	12.71 ± 4.25 (1) 12.71 ± 2.98 (2)	
			15–16 [20]	13.61 ± 4.99 (1) 13.99 ± 4.78 (2)	14.81 ± 4.85 (1) 14.74 ± 4.24 (2)	
**7. Rennie et al. 2017, South Africa** [28]	The volume of sinuses was automatically calculated in the 3D reconstruction following manual segmentation using 3D Slicer (version Mac OS X 4.10.0)	480 (276,204) (including older age groups which are not	1–4 [65]	1.62 ± 1.31 [0.05–5.88]	1.57 ± 1.24 [0.03–5.81]	0.20 ± 0.28 [0.01–1.33]
			4–7 [44]	3.33 ± 1.54 [0.68–6.26]	3.33 ± 1.52 [0.44–6.80]	0.72 ± 0.68 [0.04–3.10]
			7–10 [30]	4.56 ± 2.41 [0.42–11.81]	4.68 ± 2.56 [0.59–11.59]	1.07 ± 1.22 [0.06–5.45]
			10–13 [41]	7.53 ± 3.07 [2.16–13.83]	7.82 ± 2.93 [2.42–14.65]	2.77 ± 2.03 [0.17–8.15]
			13–16 [43]	9.47 ± 4.49 [2.52–20.20]	10.20 ± 4.77 [1.90–21.18]	3.22 ± 2.71 [0.08–12.84]
			16–19 [57]	11.48 ± 4.68 [2.07–20.52]	11.43 ± 5.41 [1.25–23.95]	4.19 ± 2.75 [0.31–13.45]

		presented in this table)							
**8. Lee et al., 2020, New Zealand**[27]	The volume of sinuses was automatically calculated in the 3D reconstruction following manual segmentation using 3D Slicer (version Mac OS X 4.10.0)	65(38,27)	0–2	0.81 ± 0.69 [0.09–2.13]					
			2–4	2.76 ± 1.16 [1.00–4.43]					
			4–6	7.12 ± 3.08 [3.05–11.43]					
			6–8	7.55 ± 1.68 [4.69–10.02]					
			8–10	9.89 ± 2.62 [7.16–13.85]					
			10–12	12.86 ± 3.94 [8.46–20.05]					
			12–14	15.31 ± 3.26 [10.48–19.70]					
			14–16	18.77 ± 5.35 [7.61–27.39]					
			16–18	21.63 ± 6.49 [15.76–33.48]					
**9. Jasso-Ramírez et al., 2023, Mexico**[4]	Multiplanar reconstruction with Centricity Universal Viewer software	210 (104,106)	<5 [60]	12.33 ± 7.66	12.74 ± 7.84	2.65 ± 4.19	2.84 ± 3.25	0.79 ± 1.41	1.14 ± 2.26
			6–10 [50]	23.88 ± 8.27	23.82 ± 7.07	6.29 ± 4.69	7.08 ± 5.84	3.94 ± 3.30	4.59 ± 4.37
			11–15 [50]	35.95 ± 9.50	35.51 ± 9.09	11.19 ± 6.67	13.34 ± 10.01	10.18 ± 4.96	12.22 ± 6.52
			>16 [50]	39.80 ± 7.76	42.57 ± 8.79	14.54 ± 5.93	14.87 ± 7.91	13.38 ± 6.75	16.05 ± 16.68
**10. Yamakawa et al.,2024, Japan**[12]	Using the image analysis software Mimics Innovation Suite Medical version (version 23.0, Materialise N. V., Belgium)	137 (including older age groups which are not presented in this table)	2–4 [9]	3.72 [3.57–5.06]		0.35 [0.23–0.63]		no pneumatization	
			5–6 [13]	6.87 [4.93–7.70]		0.53 [0.22–0.78]		0.23 [0.03–0.66]	
			7–8 [12]	9.48 [7.49–11.04]		2.38 [1.05–2.80]		0.65 [0.253–1.09]	
			9–10 [11]	10.82 [9.42–12.65]		2.19 [1.77–3.50]		1.28 [0.52–1.81]	
			11–12 [13]	13.68 [10.89–15.30]		3.37 [2.83–4.12]		2.30 [0.89–2.6]	
			13–14 [12]	16.1 [11.37–21.17]		4.28 [2.36–5.79]		2.97 [0.10–4.12]	
			15–16 [14]	18.29 [16.2–18.8]		5.60 [4.28–6.78]		2.60 [1.66–3.23]	
			17–18 [13]	21.55 [19.24–23.27]		5.29 [3.65–7.85]		2.72 [1.64–5.22]	
